# Geospatial-temporal distribution of Tegumentary Leishmaniasis in Colombia (2007–2016)

**DOI:** 10.1371/journal.pntd.0006419

**Published:** 2018-04-06

**Authors:** Giovanny Herrera, Aníbal Teherán, Iván Pradilla, Mauricio Vera, Juan David Ramírez

**Affiliations:** 1 Grupo de Investigaciones Microbiológicas-UR (GIMUR), Facultad de Ciencias Naturales y Matemáticas, Programa de Biología, Universidad del Rosario, Bogotá-Colombia; 2 Grupo de Investigación COMPLEXUS, Fundación Universitaria Juan N. Corpas, Bogotá-Colombia; 3 Residente Medicina de Emergencias, Escuela de Medicina y Ciencias de la Salud, Universidad del Rosario, Bogotá-Colombia; 4 Grupo NeURos, Escuela de Medicina y Ciencias de la Salud, Universidad del Rosario, Bogotá-Colombia; 5 Ministerio de Salud y Protección Social, Bogotá, Colombia; National Institute of Parasitic Diseases, CHINA

## Abstract

**Background:**

Tegumentary Leishmaniasis (TL) is a neglected disease with worldwide distribution and considered a public health problem, especially in Latin America. In Colombia, the governmental epidemiological surveillance system (SIVIGILA) is responsible for collecting information on the presentation of cases of TL from each of the municipalities and departments. In absence of a study compiling and analyzing currently available metadata of TL in Colombia, this study describes the geospatial-temporal distribution of TL and identifies the regions of the country on which prevention measures should be established in order to control the disease.

**Methodology/Principal findings:**

This is an exploratory descriptive analysis of the distribution of TL in Colombia. Information was collected on new cases of the disease during the years 2007–2016 from the Colombian reporting system (SIVIGILA). Incidence calculations were made based on population estimates by departments and biogeographical regions. Time evolution is shown in biennial maps. A 10-year series was analyzed, showing that the Amazon region is the most affected in terms of incidence, while the Andean region has the highest number of cases with a high variability among the departments that make it up. In those departments where there is a greater reported diversity of vector species, a large number of cases was observed.

**Conclusions/Significance:**

Transmission dynamics of TL in Colombia in the past 10 years have been variable, with a greater concentration of cases in the central and southern departments. The present study contributes to improve the understanding of the patterns of distribution of TL in Colombia and can be a basis for future studies of impact evaluation of Health policies in the country and the region.

## Author summary

Colombia is among the countries with the highest number of Tegumentary Leishmaniasis cases worldwide. Despite public health efforts, and the existence of a national epidemiological surveillance system, articulated with the regional SisLeish system, the trends followed by the disease’s prevalence and incidence have not been explored. This work presents a retrospective analysis of Tegumentary Leishmaniasis in Colombia between the years 2007 and 2016, depicting the spatiotemporal distribution of the disease throughout the Colombian territory. The results show a sustained transmission rate, especially in the Amazon and Orinoco regions, and high intraregional variability that could be attributed to each region’s characteristics (vector species diversity and low adherence to preventive measures). The Amazon region is of particular importance due to its direct connection to other endemic countries such as Peru and Brazil. This connection highlights the need to prioritize the implementation of control and prevention strategies in this region. The surveillance system displays multiple flaws, which contribute to under-reporting and have a direct impact on the regional system. Strengthening the system should become a key public health objective in the country. The results of this research can contribute to the understanding of the transmission dynamics of Leishmaniasis in Colombia, as well as to the bolstering of public policy efforts in towards prevention and control of the disease.

## Introduction

Leishmaniasis, considered one of the most neglected diseases worldwide, is a grouping of parasitic pathologies caused by protozoans of the *Leishmania* genus, and transmitted to humans primarily by insects of the Psychodidae family [1]. Leishmaniasis displays a wide range of manifestations and clinical forms of which the most common, both worldwide and in the American continent, is Cutaneous Leishmaniasis (CL) characterized by ulcerative skin lesions [1–3]. CL is considered a widespread public health issue with an elevated prevalence, considered endemic in 98 countries, adding up to a population at risk of over 350 million people. Meanwhile, Mucocutaneous Leishmaniasis (ML) affects a smaller percentage of the population, producing disfiguring lesions which compromise both the oral and nasal mucosae [1, 2, 4]. In the clinical practice, CL and ML are known as Tegumentary Leishmaniasis (TL). The American continent constitutes a special scenario for TL, given that the biological and clinical complexity of the disease is compounded by the presence of sociodemographic variability, geographical diversity, and the presence of internal armed conflicts in different countries. These features facilitate the parasite’s spread in the Americas, now present in 20 countries, of which 18 are endemic with a yearly average of 56,262 cases between 2001 and 2015. Seventy percent of the cases reported in 2015 were found in just three countries: Brazil, Colombia and Peru [3, 5, 6].

Colombia is one of the 6 countries that account for over two thirds of TL cases worldwide [7]. This clustering of the disease has been associated with various circumstances, including the presence of armed conflict, which leads to internal displacement and increases the probability of contact between vectors, reservoirs and hosts. This occurs in both the civilian and military populations. Additionally, social inequality, the absence of preventive measures in rural areas, deforestation, and geographical diversity allow the circulation of a wide array of species of both vector and parasite in up to 30 of the 32 departments in the country [3, 6, 8]. It is worth noting that Colombia is the country with the greatest number of circulating species in the world, 10 in total (*L*. *panamensis*, *L*. *braziliensis*, *L*. *guyanensis*, *L*. *infantum chagasi*, *L*. *mexicana*, *L*. *lainsoni*, *L*. *amazonensis*, *L*. *colombiensis*, *L*. *equatoriensis*, *L*. *naiffi*), and that the most prevalent species exhibit high genetic diversity [9–11]. This set of conditions conspires to produce an incidence of 33.6 per 100,000 inhabitants in 2015, leading to the categorization of the country as one of Intense Transmission by the Panamerican Health Organization (PAHO) according to its Leishmaniasis Compound Index [3]. Considering the particular characteristics found in the country, it is important that robust epidemiological surveillance models be implemented, which can detect and categorize cases not only by geographical location, but also by species. These models would allow for the execution of prevention and control strategies in order to gradually reduce the burden of disease in the country.

Currently, public health authorities in Colombia collect data on various diseases by means of a system called SIVIGILA, which gathers data on a variety of illnesses that are of public health concern [12]. In spite of the existence of this system, it is presumed that inadequate reporting is the norm in rural areas with no direct access to information systems. Likewise, and in response to the challenges around surveillance and control of leishmaniasis throughout the continent, the Regional Leishmaniasis program of the PAHO, along with representatives from six endemic countries, created a regional information system called SisLeish. The program gathers data from the epidemiological surveillance systems in each country, continually evaluating the disease’s distribution and producing regional indicators [13].

Despite the existence of information systems which compile data on TL, the geospatial and temporal distribution of the disease throughout the Colombian territory has not yet been studied. The trends and changes in TL’s distribution for recent years remains unknown. Therefore, this study aimed to analyze the spatiotemporal distribution of TL in Colombia between the years 2007 and 2016, providing a quantitative characterization of the disease’s behavior and spread in the country. Using the current available information, we also constructed a descriptive model which estimates the standardized incidence ratio in each of the departments and regions in each time period. This analysis allows us to formulate hypotheses regarding the areas most in need of attention from public health authorities.

## Methods

### Ethics statement

We report a geospatial analysis of TL data in Colombia. The data were readily obtained from existing public access databases (SIVIGILA). Hence, there are no specific ethical considerations.

### Data collection and manipulation

**Fig 1:**
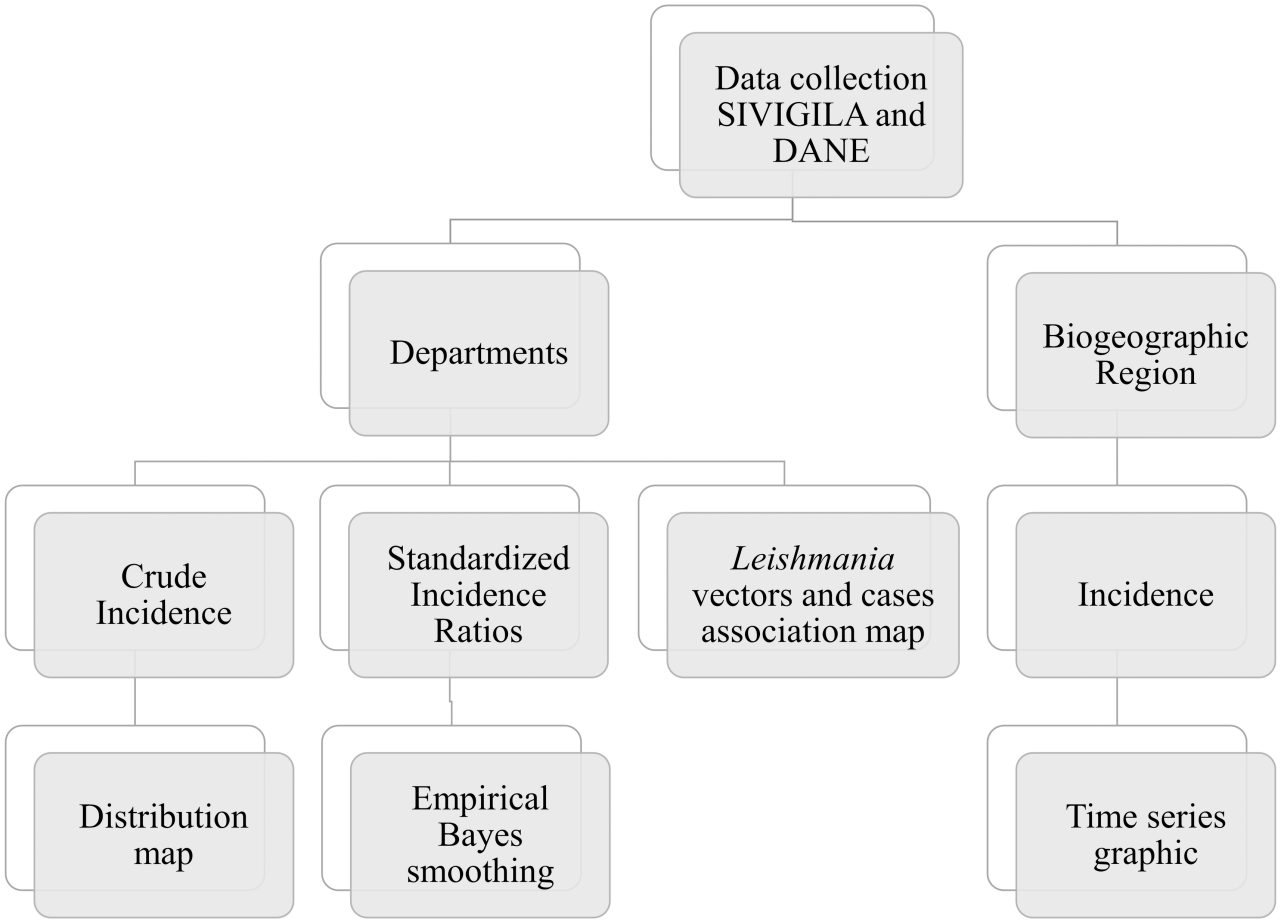
Data workflow. After collection, the data was segregated into departments, and biogeographical regions to conduct analyses at different scales. The figure shows the workflow employed for the analyses of the data retrieved.

The governmental surveillance system in Colombia is carried out by the National Public Health Surveillance System (SIVIGILA), regulated in 2006 by the office of the President of the Republic of Colombia, which is tasked with the information collection from Primary Data Generating Units (UPGD). These units correspond to Institutions Providing medical Services (IPS) in which cases of the various diseases that require mandatory reporting (Common source and transmissible events, Non transmissible prevalent diseases and Avoidable mortality events) are detected [12, 14]. Mandatory reporting events are logged in SIVIGILA’s website, and the data for TL was used to construct summary tables by municipality and department for every year from 2007 to 2016. These tables were then complimented with demographic data from the national statistical service (DANE) presented in the “Estimación y proyección de población nacional, departamental y municipal total por área 1985–2020” report [15]. The data was organized with the Microsoft Excel software in its 2016 version. The data for each geographical unit was collated both annually and biennially. Additionally, departments were grouped by geographical regions (Andean, Amazon, Caribbean, Pacific, Orinoco, and Insular) (See S1 Text for more information about geographical regions). Then, inferences were conducted regarding the geospatial distribution. The data collection and analysis process are depicted in Fig 1.

### Incidence data analysis

Descriptive statistics were used to summarize the data. Preliminarily, the crude incidence (Io) was calculated by dividing the number of cases by the projected population for each of the scales and each one of the years considered. The data were then grouped, and Io was calculated for biennial periods. Io by department was split in quartiles and graphed, using a different color for each quartile. Maps depicting incidence quartile for each department were generated from an aggregation of data from biennial periods from 2007 to 2016. Administrative polygons for Colombia were obtained from the Global Administrative Areas database, managed by the University of California Berkeley [16]. Regional polygons were constructed using R version 1.0.136 [17], and packages sp and rgdal [18, 19]. The maps were generated using QGIS version 2.18.7 Las Palmas [20]. Standardized Incidence Ratios (SIR) were calculated from the total number of cases in Colombia for the period considered using Empirical Bayes smoothing method described by Clayton and Kaldor [21].

In order to visualize the detail of the annual tendencies for each of the studied regions, a time series graphic was generated and smoothed by means of Lowess (Locally-Weighted Scatterplot Smoother, 0.3 degrees, 2 steps) using Minitab v. 18. Additionally to visualize cases and vector distributions, total case data was overlaid with vector species data reported by Ferro et al. [8] on which accumulated cases in all study periods were added. The map was constructed displaying the number of cases detected in each department for all the periods, with circles centered on each department’s centroid and radii proportional to the number of cases.

## Results

### Behavior of TL occurrence and incidence

Generally, it was observed that TL has had a stable behavior in the country in the past few years, with a stable incidence between 9.99 and 34.17 cases per 100,000 inhabitants. Closer examination of TL behavior by geographical region shows that the Amazon region exhibits the highest incidence rates for all observed periods. Incidence in this region varies from 76.75 to 240.93 cases per 100,000 inhabitants. The region with the lowest incidence is the Insular region, in which only one case occurred during the periods examined. Likewise, the Caribbean region displayed incidences between 5.11 and 13.64 cases per 100,000 inhabitants. The Andean region had the highest number of cases for all periods, reaching a total of 12,847 cases between 2009 and 2010. The Orinoco region displayed the lowest case number, with 1031 cases for the 2007–2008 period (S1 Table).

The behavior of TL by departments was highly variable. The Antioquia department presented a highest number of cases in all the time periods, registering 20,951 in total across all years studied, with an average between 1000 and 3000 cases each year. The Meta department displayed the second highest number of TL cases in the country, reaching a peak of 4019 cases in 2009, this considered as the highest number of cases in a single department in any of the studied periods. The departments with the fewest cases were Bogotá with 37 cases and San Andrés with 1 case in all the periods (S2 Table).

### Tegumentary Leishmaniasis intraregional and interdepartmental variation

**Fig 2:**
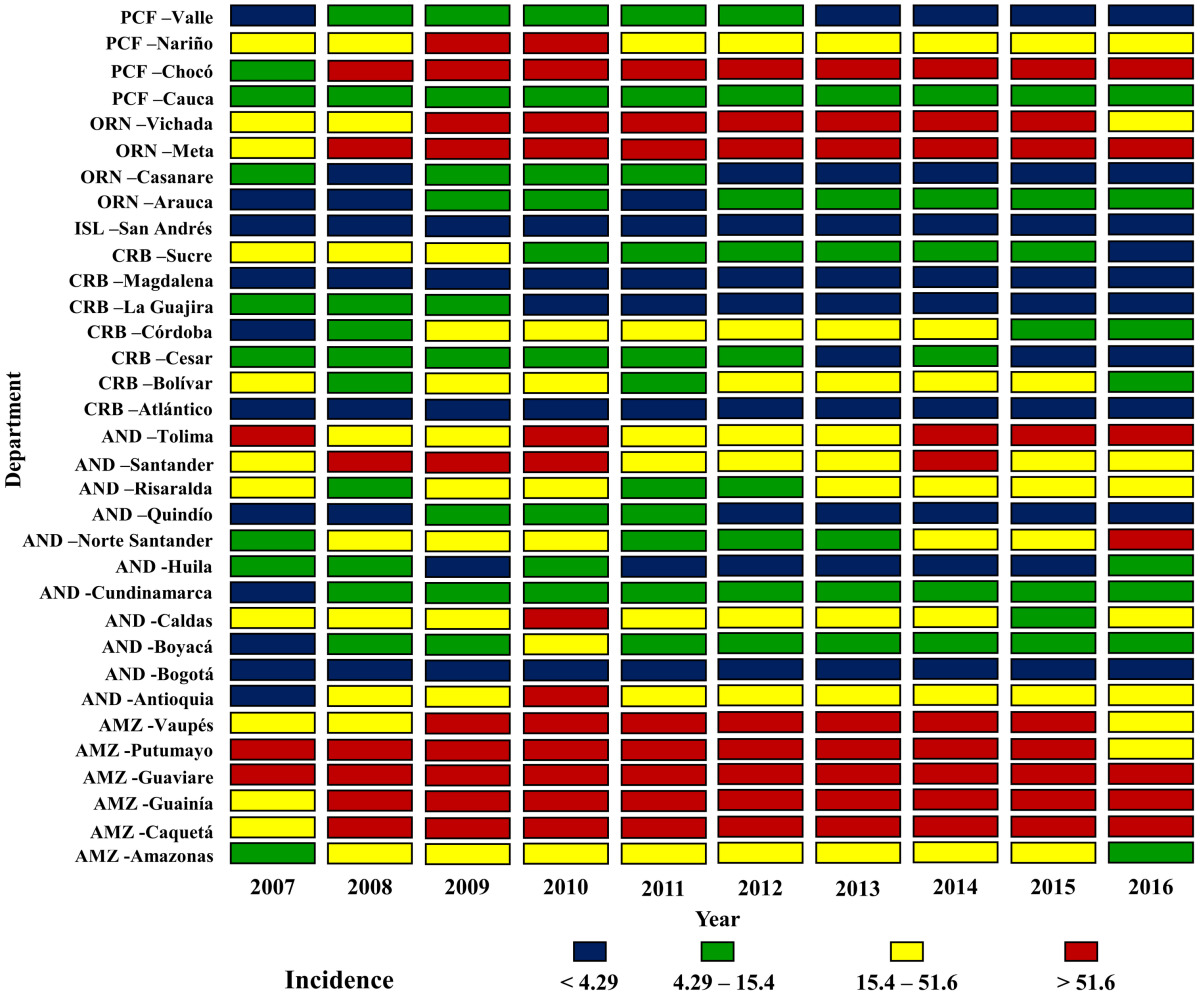
Tegumentary Leishmaniasis intraregional and interdepartmental incidence variation. Incidence (100,000 inhabitants) in the period studied was divided in quartiles and each one was represented in discrete colors; the regions were identified by abbreviations: **PCF** Pacific, **ORN** Orinoco, **ISL** Insular, **CRB** Caribbean, **AND** Andean and **AMZ** Amazon.

Fig 2 shows intraregional and interdepartmental variations in annual incidence. Across the study periods, high Io’s (>51.6 cases per 100,000 inhabitants) occurred in departments of the Amazon and Orinoco regions. Despite presenting high Io’s at the starting and ending periods, the departments in the Andean region presented mostly high to intermediate incidences (15.4–51.6 cases per 100,000 inhabitants). Departments in the Pacific and Caribbean regions displayed intermediate Io’s in 8 out of the 10 years considered. The Caribbean region displayed a greater frequency of low Io’s (<3.92 cases per 100,000 inhabitants) in later time points. Departmentally, Putumayo, Guaviare, Guainía, Caquetá, Vaupés, Meta, Chocó, and Vichada showed high Io’s for most periods considered (Fig 2).

### Geospatial distribution of TL

**Fig 3:**
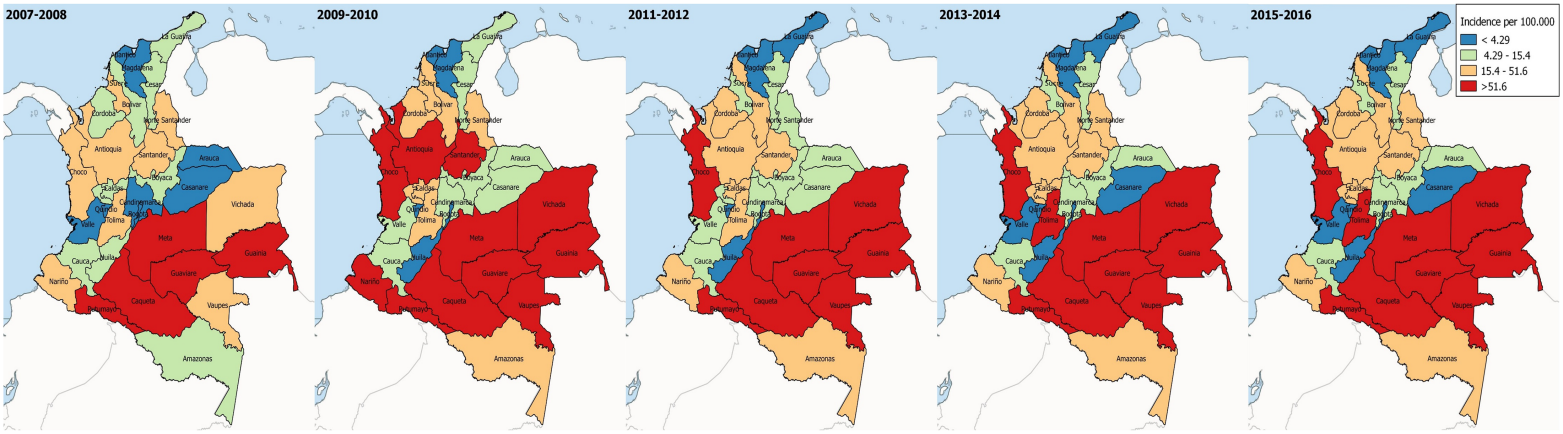
Geospatial distribution of Tegumentary Leishmaniasis in Colombia. The maps show crude biennial incidence by departments.

Maps in Fig 3 display biennial incidence divided in quartiles across departments in a choropleth format. Departments in the Amazon and Orinoco regions overall display the highest incidences, with values above the third quartile. The segmentation of the data by quartiles shows that the largest values are extreme, with a much larger range in the last group of incidences. Some departments in the Andean region, such as Antioquia, Santander, and Tolima, showed intermediate to high incidences for all time periods, while departments such as Valle and Quindío showed intermediate to low incidences.

### Standardized incidence ratios (SIR)

**Fig 4:**
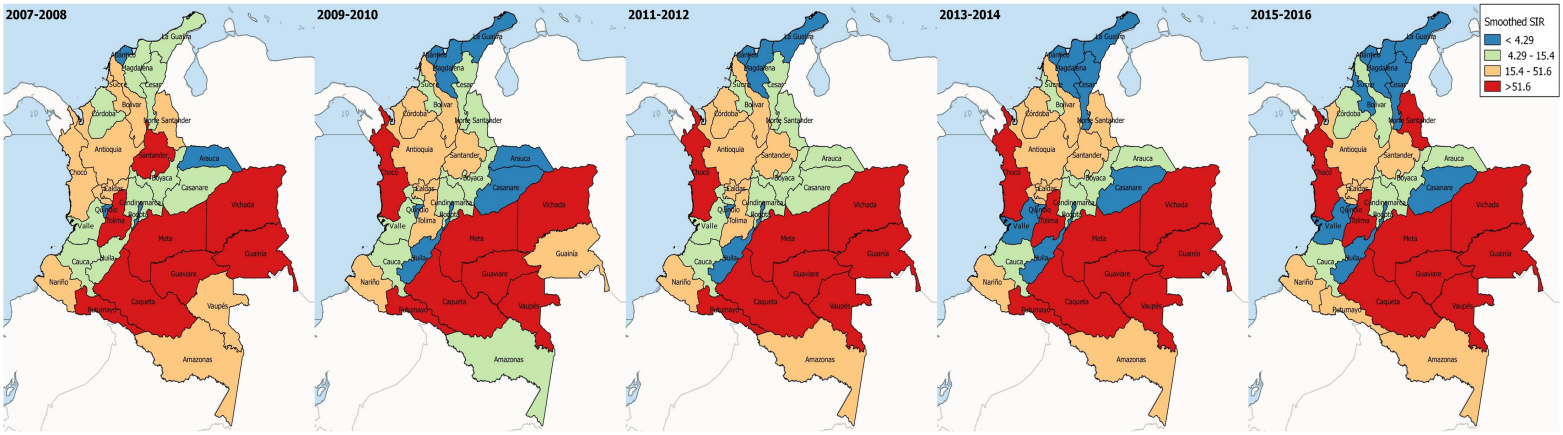
Geospatial distribution of the standardized incidence ratio of Tegumentary Leishmaniasis. Maps show the smoothed standardized incidence ratios of TL in biennial periods.

SIR showed high incidences in departments belonging to the Orinoco and Amazon regions in all the time periods. The Caribbean region displays the lowest SIRs across all time periods. Centrally-located departments show stable incidences across time periods, with the notable exceptions of Norte de Santander and Tolima, which trend towards higher values (Fig 4).

### Vector species distribution and TL cases

**Fig 5:**
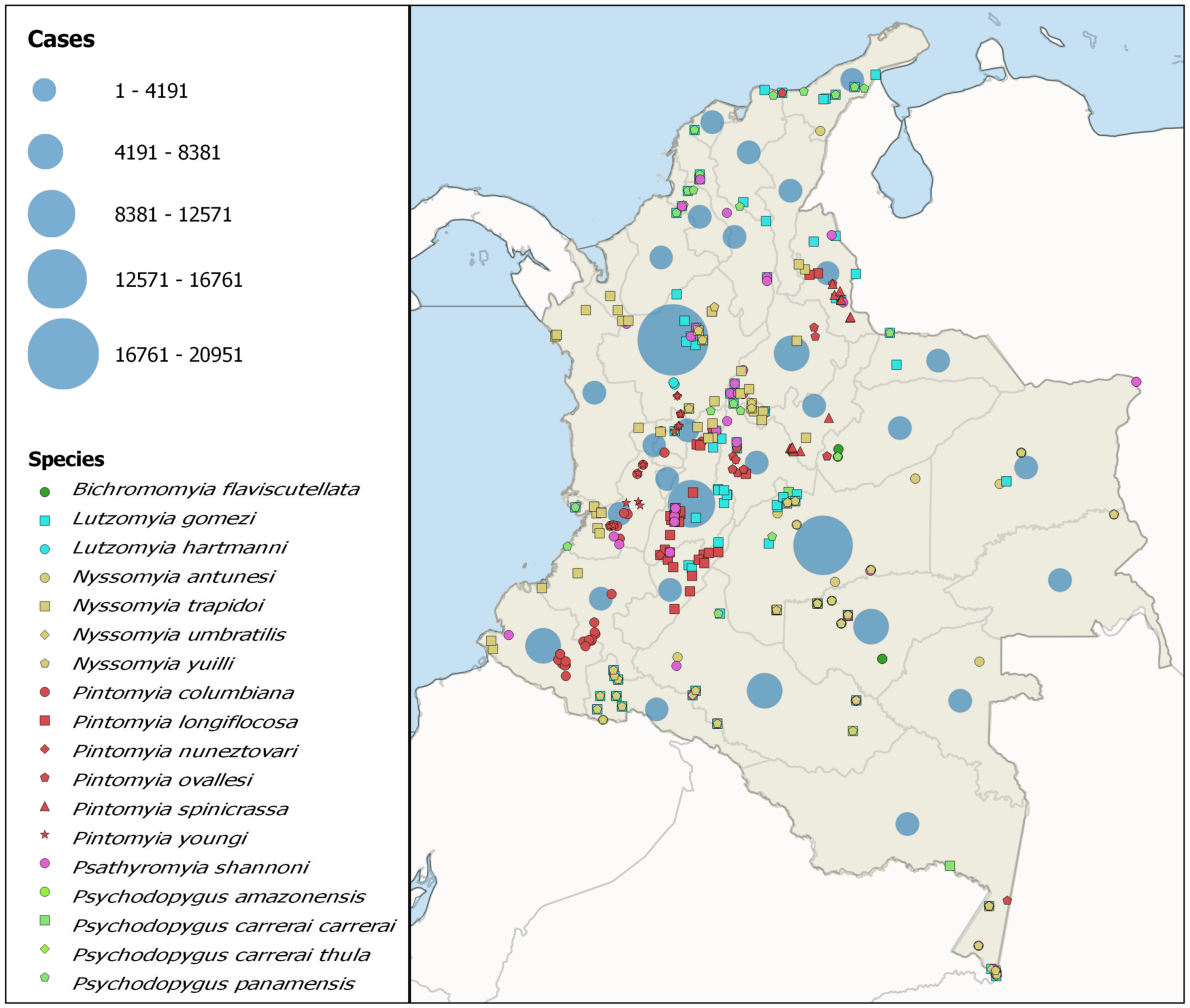
TL cases and *Leishmania* vectors association map. The map shows overlap between vector diversity and total number of departmental cases across study periods.

Superposition of vector species and leishmaniasis cases shows that departments with greater reported vector diversity tend to display an elevated number of cases, particularly in the departments belonging to the Andean and Orinoco regions (Fig 5). *Lutzomyia gomezi* and *Psychodopygus panamensis* are present in 21 departments each, considered the most widely spread species in the country. They are followed by *Psathyromyia shannoni* and *Nyssomyia trapidoi*, which are present in 16 and 11 departments, respectively. Antioquia showed the highest number of vector species, 11 overall, followed by Meta, Caldas, Boyacá, and Amazonas, with 9 species each.

### Annual tendency for TL

**Fig 6:**
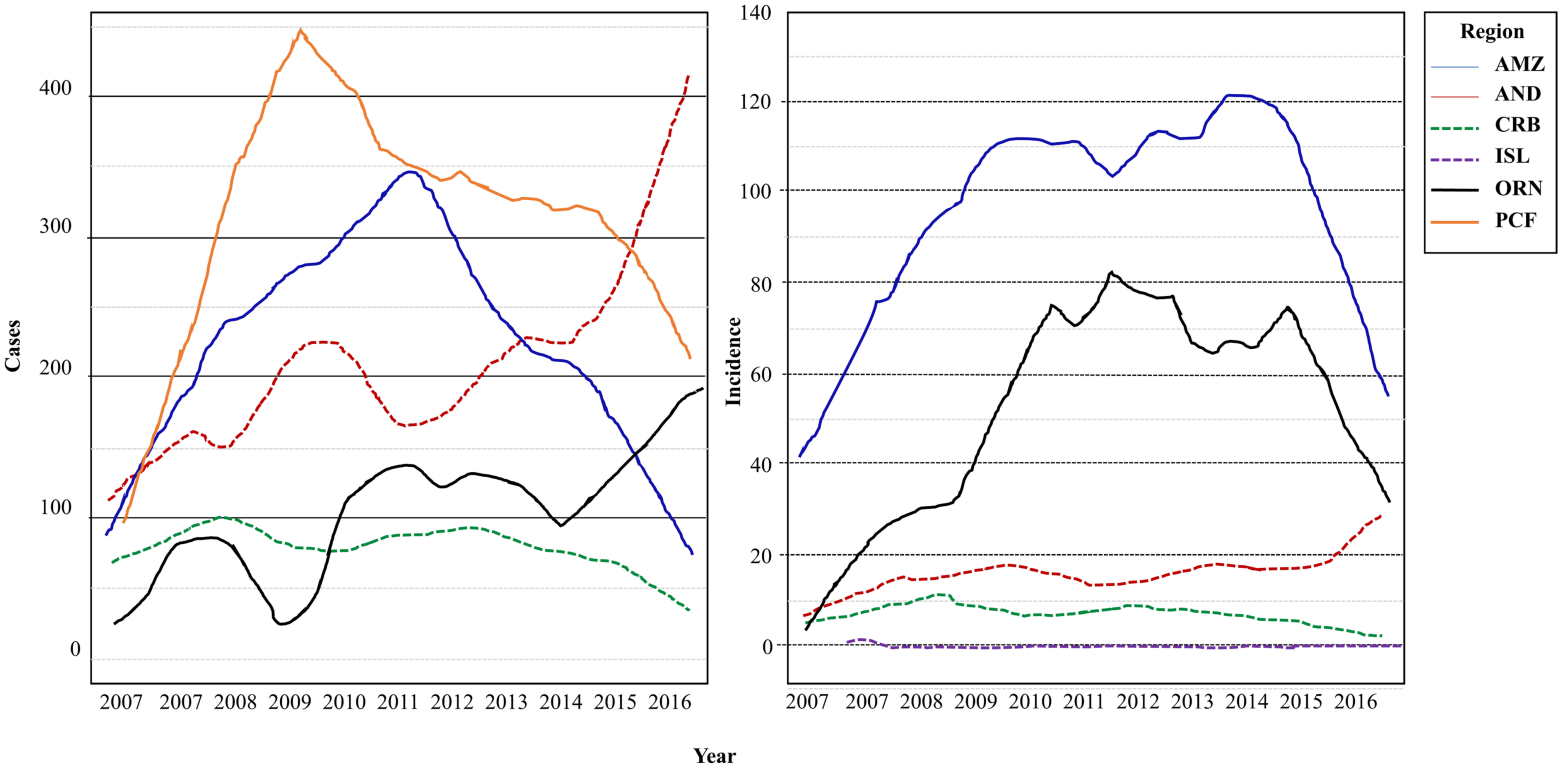
Occurrence and incidence of Tegumentary Leishmaniasis across regions. LOWESS fit was used to smoothen the frequency of Leishmaniasis (cases, incidence) by regions, in a time series graph; the dotted lines (AND, CRB, ISL) represent regions with lower incidences (100,000 habitants).

Yearly tendency of TL varied according to the region under study, showing a stable pattern in the Andean, Caribbean and Insular regions. The Amazon, Orinoco, and Pacific regions displayed a progressive increase in incidence up to the year 2010, followed by a stable behavior, mainly in the Amazon and Orinoco regions, and finally decreasing from the year 2014 to 2016. Case behavior in relation to Io backs the likely influence of the population base in the annual tendency of the event (Fig 6).

## Discussion

TL remains a public health issue in Colombia and worldwide. This study, shows that the disease trends have been stable in the past decade, particularly in the Caribbean and Insular regions (Figs 2–4 and 6). Both regions are adjacent to the Caribbean sea, with high average temperatures, and frequent droughts, which inhibit vector spread [8]. The Andean region showed an increasing tendency, which might be due to a variety of factors, including outbreaks, and changes which facilitate the infection’s permanence, urbanization, and deforestation [22–26]. The Orinoco, Amazon and Pacific regions displayed an increasing tendency during the first years considered, with a decrease in incidence in the later years (Fig 6). The initial tendency coincides with the inception of the epidemiological vigilance system, which could have altered the behavior artificially. Additionally, the geographical characteristics in these regions allow for the optimal development of vectors, thus facilitating the disease’s transmission and permanence [8].

On an intraregional level, we observed highly fluctuating incidences, mainly in the inner Andean Region and the Orinoco region, where low incidences in departments such as Huila, Quindío, Arauca and Casanare can be contrasted with the high incidences found in Santander, Tolima, Meta, and Vichada (Figs 2–4). These large differences among departments belonging to the same regions, some of which are contiguous, shows that individual departmental characteristics modify disease burden. The Andean region, which gathers over 50% of the country’s population, shows great socioeconomic diversity and highly variable geography [27]. These features have been studied extensively in previous investigations, and have demonstrated a strong influence on disease presentation, particularly in those region in which the biogeographical and social conditions allow not only for vector development, but also for its propagation due to deforestation and urbanization [28, 29]. Another important aspect is the degree of sophistication of the individual departmental reporting systems, which has a direct impact not only on diagnosis, but also on the disease’s treatment. This can ultimately result in an increase in risk to all the population, since some of the population inhabits distant areas where it is difficult to diagnose and treat them, thus becoming reservoirs and perpetuating the disease’s cycle [28].

On the other hand, the Amazon region demands special attention, not only from Colombia, but also from other countries in the region, due to its connections with countries as Brazil and Peru, which allow for the continued transit of potentially infected humans, and the continuity across borders of vector habitats [3]. This was one of the most affected areas by the disease, with high incidences across all study periods. This can be attributed to the wealth of vector species and of mammals that serve as disease reservoirs, which helps maintain the epidemiological circuit and, in turn, transmission rates [8]. The population in this region is relatively low, when compared with other regions with similar transmission rates (S1 Table), due in part to the large percentage of the territory occupied by tropical rainforest. The opposite situation is seen in the Andean region, which is made up of the 11 departments that make up the majority of the country’s population, in which the large number of cases does not result in elevated incidences (Figs 1 and 5; S1 Table). Some of the factors that may have produced these population concentrations are the armed conflict that was active during the studied periods, and a lack of public policies that favored the agricultural economic sector, which combined led to a reduction in rural population growth, mass displacement from rural to urban areas and an increase in internal migration [30, 31]. It is worth noting that some departments in the Orinoco and Amazon regions, have shown high transmission rates and prevalence in independent studies [9, 32]. These same departments report fewer cases to the central surveillance system than departments in other regions with more robust healthcare infrastructure. These same areas are often the most accessible to research groups, which strengthens the surveillance network, particularly in the Andean region.

Likewise, the great diversity of circulating *Leishmania* species in Colombia plays a fundamental role in the permanence and propagation of the disease, given that neither epidemiological surveillance, nor treatment, take this variable into account. This assumes that all species are equally susceptible to treatment, which has been refuted in multiple studies [4, 11, 33–35]. The presence of some vectors in over half of the departments in Colombia (Fig 5) shows the importance of this factor in the perpetuation of the epidemiological cycle. This underlines the need to address this issue in order to control Leishmaniasis in the country [8, 36]. Likewise, the superposition of the vector species with the TL cases is observed most often in zones with high incidences, reinforcing the importance of the vector’s role (Fig 6). Studies carried out on vectors in Mexico and Spain have shown similar behaviors to those herein presented and have highlighted the need to analyze not only the distributions of the species, but also the bio-climatic and sociodemographic factors as transcendental elements in the transmission of the disease [37, 38]. Likewise, studies conducted in Brazil have associated factors such as urbanization and poor sanitary conditions with the adaptation and maintenance of the vector life cycle [39, 40]. This highlights the importance of complementing the epidemiological surveillance with the phlebotominae analysis, more when Ferro et al., shows through predictive analysis the possible dispersion of each of the species of the vectors involved in the transmission, where it is evident that almost throughout of all the Colombian territory and especially in bordering zones, there can be presence of diverse species of sandfly vectors [8]. The above, denotes the priority that the strengthening of the Epidemiological Surveillance System should have, not only at a national level, but also at a regional level, since the joint analysis of the different elements that make up the epidemiological circuit of the disease will allow not only a better understanding of the system, but also establish more efficient prevention and control measures.

Despite the multiple efforts undertaken by governmental agencies to control the disease, many characteristics of the surveillance system must be expanded on to be able to formulate public policies that lead to a reduction in case presentation. Retrospective studies play a significant role in this process, since trends can be identified, and then prevention and control measures can be formulated. Of note, studies in Brazil and Iran have evaluated the spatial distribution and epidemiological characteristics of leishmaniasis, becoming the starting point for a more effective allocation of resources, guiding national and regional public health policy [41, 42]. Despite the existence of a reporting system, underreporting is common in the most remote rural areas. These areas coincide with areas highly affected by armed conflict, which makes it challenging to estimate the real number of cases with certainty. It is expected that the signing of the peace accords in Colombia will lead to a more sustained and widespread governmental presence in these areas, allowing for more reliable information gathering in both the civilian population and in the population belonging to illegal armed groups. The weaknesses of the national epidemiological surveillance system have direct repercussions over the Regional SisLeish system [13], which collates data from all countries in the Americas in order to guide the efforts of the PAHO. Non-adherence to the surveillance system and delays in reporting have been detected in the SisLeish system, and can be traced back directly to local systems [13]. The data presented here differ significantly from those reported by Maia-Elkhoury et al. [43] with regards to incidence. This is due to differences in the definition of the population at risk. This study considered the entire population of the department as being at risk, instead of just accounting for the population of municipalities in which cases were reported. Given that underreporting is a major hurdle to analysis, we considered that the totality of the population should be considered at risk, instead of the population of the few municipalities which report cases to the national system. The case of the Amazonas department is illustrative, where some of the municipalities report significant numbers of cases for some years, and none for others (Fig 2). The population cannot be considered as not having been at risk during the years when no cases were reported. Differences may also have arisen due to base populations being different. The base populations considered in our analysis were extrapolated from census projections of 2005, since current population dynamics are unknown.

The SIR model allows us to highlight departments that are at particularly high risk, and which merit increased attention from the authorities. Although more sophisticated modeling techniques were attempted, high interdepartmental variability in the occurrence of cases resulted in inadequate fits. Geo-political changes introduced further variability, which could correspond to a high rate of internal migration. It should be noted that the highest rates of internal migration during the period studied were found in the departments of Boyacá, Cundinamarca, Meta, and the Orinoco and Amazon regions, which would reflect the displacement of potentially infected populations to urban areas [29, 44–46]. It is important to note that variables that could aid in modelling, like deforestation and socioeconomic characteristics, are not reported directly by SIVIGILA, and can have dramatic effects on disease occurrence [25, 38].

Disease dynamics may change significantly in the future due to historical transitions that are underway. The resolution of the armed conflict with the FARC guerrilla, and the establishment of rural transitional zones, will likely lead to many former combatants to be detected by the epidemiological surveillance system. Public health agencies should prepare not only for a significant increase in the number of resources required for diagnosis and treatment, but also for the redistribution of TL cases in the country, which will require changes in resource allocation. This abrupt change may lead to case number spikes in some areas previously considered as low transmission rate areas, which do not correspond to outbreaks, but which will be detected as such by the current system. In spite of SIVIGILA’s shortcomings, it is worthwhile noting that the system must be strengthened and extended, since the data collected by the system are the basis for the analysis of the country’s health situation, giving birth to all of the national public health initiatives.

In conclusion, to our knowledge, this is the first study analyzing the TL epidemiological situation for a 10-year period in Colombia, accounting for all geographical subdivisions, and vector species distribution in the country. The Andean, Amazon, and Orinoco regions account for over 75% of the cases of TL in Colombia, so that public health efforts should be extended there, particularly away from urban areas. Enhanced control can be achieved through the implementation of disease vigilance and control systems, and the strengthening of public health networks that could reduce the disease’s impact on the population. Adequate geospatial modelling of the disease could be achieved by means of a more extensive case description which includes socioeconomical and biogeographical data. This information would be required in order to develop predictive models that could more accurately guide public health efforts towards this neglected disease.

## Supporting information

S1 Text Natural regions of Colombia. (DOCX)

S1 Table Biannual Tegumentary Leishmaniasis per biogeographical region. (DOCX)

S2 Table Tegumentary Leishmaniasis cases per department. (DOCX)
