# Supplementary material for: Geospatial-temporal distribution of Tegumentary Leishmaniasis in Colombia (2007–2016)
Source: PLoS Negl Trop Dis. 2018 Apr 6;12(4):e0006419. doi: 10.1371/journal.pntd.0006419 (PMC5906026; doi:10.1371/journal.pntd.0006419)
Supplement: S1 Text — (DOCX) [file pntd.0006419.s001.docx]

**S1 Text**

**Natural Regions of Colombia**


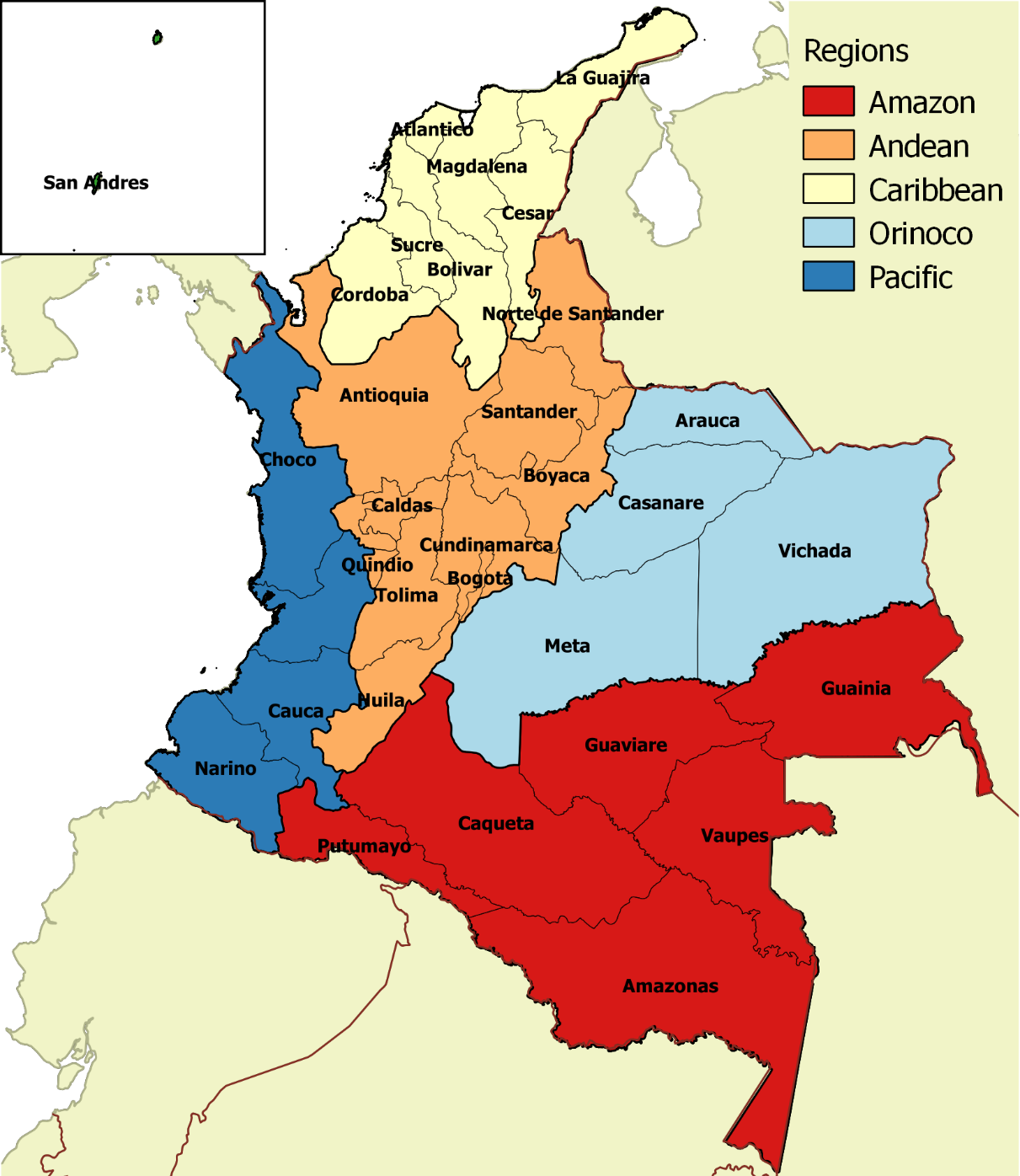


**Valle**

Colombia is a country with great geographical, ethnic, cultural and socioeconomic diversity. However, the country can be grouped into six natural regions according to their climate, terrain, and predominant ecosystems. This division is not purely geographical, and there is considerable overlap with the groupings defined by governmental budget allocation.

**Insular Region**

| **Region** | **Year** | **Population** |
| --- | --- | --- |
| **Insular** | 2007 | 71,613 |
|  | 2008 | 72,167 |
|  | 2009 | 72,735 |
|  | 2010 | 73,320 |
|  | 2011 | 73,925 |
|  | 2012 | 74,541 |
|  | 2013 | 75,167 |
|  | 2014 | 75,801 |
|  | 2015 | 76,442 |
|  | 2016 | 77,101 |

The smallest region in the country, it encompasses the San Andres archipelago. Due to its climate and sea-level altitude, TL-transmitting vectors are not found there, which is why reported cases very likely correspond to subjects who acquired the disease elsewhere in the country (1).

**Caribbean Region**

| **Region** | **Year** | **Population** |
| --- | --- | --- |
| **Caribbean** | 2007 | 9,276,035 |
|  | 2008 | 9,407,456 |
|  | 2009 | 9,540,029 |
|  | 2010 | 9,674,097 |
|  | 2011 | 9,810,720 |
|  | 2012 | 9,948,437 |
|  | 2013 | 10,087,002 |
|  | 2014 | 10,226,146 |
|  | 2015 | 10,365,577 |
|  | 2016 | 10,506,836 |

The Caribbean region is made up of the northern departments: Atlántico, Bolívar, Cesar, Córdoba, La Guajira, Magdalena, and Sucre. The geography is dominated by coastal plains and mountain ranges, with tropical rainforest and dry forest ecosystems (1).

**Amazon Region**

| **Region** | **Year** | **Population** |
| --- | --- | --- |
| **Amazon** | 2007 | 991,993 |
|  | 2008 | 1,004,087 |
|  | 2009 | 1,016,439 |
|  | 2010 | 1,029,002 |
|  | 2011 | 1,041,778 |
|  | 2012 | 1,054,782 |
|  | 2013 | 1,068,026 |
|  | 2014 | 1,081,518 |
|  | 2015 | 1,095,273 |
|  | 2016 | 1,109,282 |

The Amazon region boasts great diversity in both fauna and flora species. The tropical rainforest is the predominant biome, with warm weather and abundant precipitation. The human population is predominantly indigenous and despite covering a large percentage of the country’s territory, it is the second least populated region. It is located in southern Colombia, comprising the departments of Amazonas, Caquetá, Guainía, Guaviare, Putumayo and Vaupés (1).

**Andean Region**

| **Region** | **Year** | **Population** |
| --- | --- | --- |
| **Andean** | 2007 | 24,584,595 |
|  | 2008 | 24,858,147 |
|  | 2009 | 25,132,296 |
|  | 2010 | 25,406,439 |
|  | 2011 | 25,681,022 |
|  | 2012 | 25,955,416 |
|  | 2013 | 26,229,526 |
|  | 2014 | 26,503,251 |
|  | 2015 | 26,776,508 |
|  | 2016 | 27,049,759 |

The Andean Region covers a third of Colombia’s territory and is home to roughly half its population. The region contains the northernmost portion of the Andes, which gives rise to three lesser mountain ranges. These ranges account for the high climatic variability observed within the region, with altitudes that range from sea-level to over 5000m. The departments of Antioquia, Bogotá D.C., Boyacá, Caldas, Cundinamarca, Huila, Norte de Santander, Quindío, Risaralda, Santander and Tolima are found here (1).

**Orinoco Region**

| **Region** | **Year** | **Population** |
| --- | --- | --- |
| **Orinoco** | 2007 | 1,422,550 |
|  | 2008 | 1,450,834 |
|  | 2009 | 1,479,195 |
|  | 2010 | 1,507,683 |
|  | 2011 | 1,536,330 |
|  | 2012 | 1,565,095 |
|  | 2013 | 1,593,972 |
|  | 2014 | 1,622,952 |
|  | 2015 | 1,652,019 |
|  | 2016 | 1,681,273 |

The Orinoco region contains a significant portion of the larger Orinoco basin. A large number of rivers crisscross its territory, and warm tropical and subtropical forests are the predominant ecosystems. Sparsely populated, it comprises the departments of Arauca, Casanare, Meta and Vichada (1).

**Pacific Region**

| **Region** | **Year** | **Population** |
| --- | --- | --- |
| **Pacific** | 2007 | 7,579,248 |
|  | 2008 | 7,657,569 |
|  | 2009 | 7,737,064 |
|  | 2010 | 7,817,664 |
|  | 2011 | 7,899,921 |
|  | 2012 | 7,983,101 |
|  | 2013 | 8,067,077 |
|  | 2014 | 8,151,700 |
|  | 2015 | 8,236,798 |
|  | 2016 | 8,323,381 |

It is one of the most humid regions worldwide and is covered by tropical rainforests with great biodiversity. It is bordered by the Pacific Ocean and the West Andes mountain range. Although rich in natural resources, the region shows scarce urban development and infrastructure. The departments of Cauca, Chocó, Nariño and Valle can be found here (1).

1. INSTITUTO GEOGRAFICO AGUSTIN CODAZZI 2018 [Available from: <http://www.igac.gov.co/geoportal>.
