# Supplementary material for: Geospatial-temporal distribution of Tegumentary Leishmaniasis in Colombia (2007–2016)
Source: PLoS Negl Trop Dis. 2018 Apr 6;12(4):e0006419. doi: 10.1371/journal.pntd.0006419 (PMC5906026; doi:10.1371/journal.pntd.0006419)
Supplement: S1 Table — (DOCX) [file pntd.0006419.s002.docx]

**S1 Table. Biannual Tegumentary Leishmaniasis per biogeographical region**

| **Region** | **Year** | **Cases** | **Incidence/100000** |
| --- | --- | --- | --- |
| AMAZON | 07-08 | 2300 | 115.23 |
|  | 09-10 | 4928 | 240.93 |
|  | 11-12 | 4355 | 207.72 |
|  | 13-14 | 2686 | 124.96 |
|  | 15-16 | 1692 | 76.75 |
| ANDEAN | 07-08 | 6317 | 12.78 |
|  | 09-10 | 12847 | 25.42 |
|  | 11-12 | 7473 | 14.47 |
|  | 13-14 | 10011 | 18.60 |
|  | 15-16 | 6913 | 21.63 |
| CARIBBEAN | 07-08 | 1348 | 7.21 |
|  | 09-10 | 2620 | 13.64 |
|  | 11-12 | 1808 | 9.15 |
|  | 13-14 | 1953 | 9.61 |
|  | 15-16 | 1066 | 5.11 |
| INSULAR | 07-08 | 1 | .70 |
|  | 09-10 | 0 | .00 |
|  | 11-12 | 0 | .00 |
|  | 13-14 | 0 | .00 |
|  | 15-16 | 0 | .00 |
| ORINOCO | 07-08 | 1031 | 35.88 |
|  | 09-10 | 5793 | 193.95 |
|  | 11-12 | 2508 | 80.87 |
|  | 13-14 | 2579 | 80.17 |
|  | 15-16 | 1939 | 58.17 |
| PACIFIC | 07-08 | 1561 | 10.24 |
|  | 09-10 | 3918 | 25.19 |
|  | 11-12 | 2782 | 17.52 |
|  | 13-14 | 2697 | 16.63 |
|  | 15-16 | 2321 | 14.02 |
