# Supplementary material for: Geospatial-temporal distribution of Tegumentary Leishmaniasis in Colombia (2007–2016)
Source: PLoS Negl Trop Dis. 2018 Apr 6;12(4):e0006419. doi: 10.1371/journal.pntd.0006419 (PMC5906026; doi:10.1371/journal.pntd.0006419)
Supplement: S2 Table — (DOCX) [file pntd.0006419.s003.docx]

**S2 Table. Tegumentary Leishmaniasis Cases per Department**

| **State** | **Region** | **Year** | **Population** | **Cases** | **Incidence/100000** |
| --- | --- | --- | --- | --- | --- |
| Amazonas | AMZ | 2007 | 69,474 | 4 | 5.76 |
|  |  | 2008 | 70,313 | 16 | 22.76 |
|  |  | 2009 | 71,167 | 16 | 22.48 |
|  |  | 2010 | 72,017 | 13 | 18.05 |
|  |  | 2011 | 72,858 | 31 | 42.55 |
|  |  | 2012 | 73,699 | 28 | 37.99 |
|  |  | 2013 | 74,541 | 16 | 21.46 |
|  |  | 2014 | 75,388 | 22 | 29.18 |
|  |  | 2015 | 76,243 | 29 | 38.04 |
|  |  | 2016 | 77,088 | 10 | 12.97 |
| Antioquia | AND | 2007 | 5,835,008 | 128 | 2.19 |
|  |  | 2008 | 5,911,330 | 1686 | 28.52 |
|  |  | 2009 | 5,988,458 | 2952 | 49.29 |
|  |  | 2010 | 6,065,846 | 3488 | 57.5 |
|  |  | 2011 | 6,143,709 | 2124 | 34.57 |
|  |  | 2012 | 6,221,742 | 2038 | 32.76 |
|  |  | 2013 | 6,299,886 | 2256 | 35.81 |
|  |  | 2014 | 6,378,069 | 2777 | 43.54 |
|  |  | 2015 | 6,456,207 | 1751 | 27.12 |
|  |  | 2016 | 6,534,764 | 1751 | 26.8 |
| Arauca | ORN | 2007 | 238,361 | 4 | 1.68 |
|  |  | 2008 | 241,446 | 7 | 2.9 |
|  |  | 2009 | 244,507 | 16 | 6.54 |
|  |  | 2010 | 247,541 | 10 | 4.04 |
|  |  | 2011 | 250,569 | 9 | 3.59 |
|  |  | 2012 | 253,565 | 17 | 6.7 |
|  |  | 2013 | 256,527 | 21 | 8.19 |
|  |  | 2014 | 259,447 | 26 | 10.02 |
|  |  | 2015 | 262,315 | 20 | 7.62 |
|  |  | 2016 | 265,190 | 18 | 6.79 |
| Atlantico | CRB | 2007 | 2,225,462 | 1 | 0.04 |
|  |  | 2008 | 2,255,164 | 0 | 0 |
|  |  | 2009 | 2,284,840 | 0 | 0 |
|  |  | 2010 | 2,314,447 | 0 | 0 |
|  |  | 2011 | 2,344,140 | 0 | 0 |
|  |  | 2012 | 2,373,680 | 0 | 0 |
|  |  | 2013 | 2,403,027 | 0 | 0 |
|  |  | 2014 | 2,432,145 | 0 | 0 |
|  |  | 2015 | 2,461,001 | 0 | 0 |
|  |  | 2016 | 2,489,709 | 4 | 0.16 |
| Bogota | AND | 2007 | 7,050,228 | 28 | 0.4 |
|  |  | 2008 | 7,155,052 | 0 | 0 |
|  |  | 2009 | 7,259,597 | 0 | 0 |
|  |  | 2010 | 7,363,782 | 0 | 0 |
|  |  | 2011 | 7,467,804 | 0 | 0 |
|  |  | 2012 | 7,571,345 | 0 | 0 |
|  |  | 2013 | 7,674,366 | 0 | 0 |
|  |  | 2014 | 7,776,845 | 0 | 0 |
|  |  | 2015 | 7,878,783 | 0 | 0 |
|  |  | 2016 | 7,980,001 | 9 | 0.11 |
| Bolivar | CRB | 2007 | 1,917,112 | 310 | 16.17 |
|  |  | 2008 | 1,937,316 | 263 | 13.58 |
|  |  | 2009 | 1,958,224 | 618 | 31.56 |
|  |  | 2010 | 1,979,781 | 489 | 24.7 |
|  |  | 2011 | 2,002,391 | 244 | 12.19 |
|  |  | 2012 | 2,025,521 | 489 | 24.14 |
|  |  | 2013 | 2,049,083 | 371 | 18.11 |
|  |  | 2014 | 2,072,976 | 409 | 19.73 |
|  |  | 2015 | 2,097,086 | 323 | 15.4 |
|  |  | 2016 | 2,122,021 | 252 | 11.88 |
| Boyacá | AND | 2007 | 1,260,854 | 43 | 3.41 |
|  |  | 2008 | 1,263,281 | 108 | 8.55 |
|  |  | 2009 | 1,265,517 | 112 | 8.85 |
|  |  | 2010 | 1,267,597 | 245 | 19.33 |
|  |  | 2011 | 1,269,401 | 109 | 8.59 |
|  |  | 2012 | 1,271,136 | 130 | 10.23 |
|  |  | 2013 | 1,272,844 | 150 | 11.78 |
|  |  | 2014 | 1,274,571 | 170 | 13.34 |
|  |  | 2015 | 1,276,367 | 80 | 6.27 |
|  |  | 2016 | 1,278,061 | 152 | 11.89 |
| Caldas | AND | 2007 | 972,590 | 146 | 15.01 |
|  |  | 2008 | 974,514 | 143 | 14.67 |
|  |  | 2009 | 976,438 | 171 | 17.51 |
|  |  | 2010 | 978,362 | 772 | 78.91 |
|  |  | 2011 | 980,281 | 216 | 22.03 |
|  |  | 2012 | 982,202 | 165 | 16.8 |
|  |  | 2013 | 984,128 | 374 | 38 |
|  |  | 2014 | 986,061 | 197 | 19.98 |
|  |  | 2015 | 988,003 | 115 | 11.64 |
|  |  | 2016 | 989,942 | 304 | 30.71 |
| Caquetá | AMZ | 2007 | 430,960 | 166 | 38.52 |
|  |  | 2008 | 436,443 | 377 | 86.38 |
|  |  | 2009 | 442,033 | 1112 | 251.56 |
|  |  | 2010 | 447,723 | 591 | 132 |
|  |  | 2011 | 453,562 | 539 | 118.84 |
|  |  | 2012 | 459,484 | 516 | 112.3 |
|  |  | 2013 | 465,477 | 357 | 76.7 |
|  |  | 2014 | 471,527 | 326 | 69.14 |
|  |  | 2015 | 477,619 | 284 | 59.46 |
|  |  | 2016 | 483,834 | 248 | 51.26 |
| Casanare | ORN | 2007 | 307,387 | 14 | 4.55 |
|  |  | 2008 | 313,433 | 7 | 2.23 |
|  |  | 2009 | 319,502 | 14 | 4.38 |
|  |  | 2010 | 325,596 | 15 | 4.61 |
|  |  | 2011 | 331,714 | 18 | 5.43 |
|  |  | 2012 | 337,858 | 11 | 3.26 |
|  |  | 2013 | 344,027 | 10 | 2.91 |
|  |  | 2014 | 350,221 | 11 | 3.14 |
|  |  | 2015 | 356,438 | 6 | 1.68 |
|  |  | 2016 | 362,698 | 9 | 2.48 |
| Cauca | PCF | 2007 | 1,287,545 | 95 | 7.38 |
|  |  | 2008 | 1,297,594 | 117 | 9.02 |
|  |  | 2009 | 1,308,090 | 108 | 8.26 |
|  |  | 2010 | 1,318,983 | 157 | 11.9 |
|  |  | 2011 | 1,330,666 | 181 | 13.6 |
|  |  | 2012 | 1,342,617 | 141 | 10.5 |
|  |  | 2013 | 1,354,744 | 139 | 10.26 |
|  |  | 2014 | 1,366,937 | 136 | 9.95 |
|  |  | 2015 | 1,379,070 | 95 | 6.89 |
|  |  | 2016 | 1,391,889 | 77 | 5.53 |
| Cesar | CRB | 2007 | 928,569 | 49 | 5.28 |
|  |  | 2008 | 941,207 | 98 | 10.41 |
|  |  | 2009 | 953,827 | 104 | 10.9 |
|  |  | 2010 | 966,420 | 48 | 4.97 |
|  |  | 2011 | 979,015 | 50 | 5.11 |
|  |  | 2012 | 991,566 | 61 | 6.15 |
|  |  | 2013 | 1,004,064 | 32 | 3.19 |
|  |  | 2014 | 1,016,503 | 60 | 5.9 |
|  |  | 2015 | 1,028,880 | 20 | 1.94 |
|  |  | 2016 | 1,041,203 | 16 | 1.54 |
| Choco | PCF | 2007 | 462,667 | 37 | 8 |
|  |  | 2008 | 467,099 | 239 | 51.17 |
|  |  | 2009 | 471,601 | 473 | 100.3 |
|  |  | 2010 | 476,173 | 455 | 95.55 |
|  |  | 2011 | 480,826 | 324 | 67.38 |
|  |  | 2012 | 485,545 | 468 | 96.39 |
|  |  | 2013 | 490,327 | 405 | 82.6 |
|  |  | 2014 | 495,171 | 434 | 87.65 |
|  |  | 2015 | 500,076 | 611 | 122.18 |
|  |  | 2016 | 505,046 | 517 | 102.37 |
| Córdoba | CRB | 2007 | 1,511,981 | 33 | 2.18 |
|  |  | 2008 | 1,535,375 | 109 | 7.1 |
|  |  | 2009 | 1,558,793 | 248 | 15.91 |
|  |  | 2010 | 1,582,718 | 707 | 44.67 |
|  |  | 2011 | 1,607,463 | 299 | 18.6 |
|  |  | 2012 | 1,632,614 | 367 | 22.48 |
|  |  | 2013 | 1,658,090 | 343 | 20.69 |
|  |  | 2014 | 1,683,792 | 485 | 28.8 |
|  |  | 2015 | 1,709,603 | 226 | 13.22 |
|  |  | 2016 | 1,736,218 | 99 | 5.7 |
| Cundinamarca | AND | 2007 | 2,358,115 | 45 | 1.91 |
|  |  | 2008 | 2,397,511 | 97 | 4.05 |
|  |  | 2009 | 2,437,151 | 208 | 8.53 |
|  |  | 2010 | 2,477,036 | 286 | 11.55 |
|  |  | 2011 | 2,517,215 | 196 | 7.79 |
|  |  | 2012 | 2,557,623 | 201 | 7.86 |
|  |  | 2013 | 2,598,245 | 191 | 7.35 |
|  |  | 2014 | 2,639,059 | 258 | 9.78 |
|  |  | 2015 | 2,680,041 | 220 | 8.21 |
|  |  | 2016 | 2,721,368 | 302 | 11.1 |
| Guainía | AMZ | 2007 | 36,464 | 17 | 46.62 |
|  |  | 2008 | 37,084 | 38 | 102.47 |
|  |  | 2009 | 37,705 | 19 | 50.39 |
|  |  | 2010 | 38,328 | 28 | 73.05 |
|  |  | 2011 | 38,949 | 26 | 66.75 |
|  |  | 2012 | 39,574 | 64 | 161.72 |
|  |  | 2013 | 40,203 | 32 | 79.6 |
|  |  | 2014 | 40,839 | 78 | 190.99 |
|  |  | 2015 | 41,482 | 52 | 125.36 |
|  |  | 2016 | 42,123 | 33 | 78.34 |
| Guaviare | AMZ | 2007 | 98,688 | 309 | 313.11 |
|  |  | 2008 | 100,208 | 816 | 814.31 |
|  |  | 2009 | 101,759 | 986 | 968.96 |
|  |  | 2010 | 103,307 | 1071 | 1036.72 |
|  |  | 2011 | 104,846 | 846 | 806.9 |
|  |  | 2012 | 106,386 | 609 | 572.44 |
|  |  | 2013 | 107,934 | 329 | 304.82 |
|  |  | 2014 | 109,490 | 585 | 534.3 |
|  |  | 2015 | 111,060 | 414 | 372.77 |
|  |  | 2016 | 112,621 | 226 | 200.67 |
| Huila | AND | 2007 | 1,040,050 | 67 | 6.44 |
|  |  | 2008 | 1,054,430 | 82 | 7.78 |
|  |  | 2009 | 1,068,820 | 39 | 3.65 |
|  |  | 2010 | 1,083,200 | 43 | 3.97 |
|  |  | 2011 | 1,097,615 | 35 | 3.19 |
|  |  | 2012 | 1,111,989 | 36 | 3.24 |
|  |  | 2013 | 1,126,314 | 12 | 1.07 |
|  |  | 2014 | 1,140,585 | 21 | 1.84 |
|  |  | 2015 | 1,154,804 | 28 | 2.42 |
|  |  | 2016 | 1,168,910 | 49 | 4.19 |
| La Guajira | CRB | 2007 | 735,974 | 47 | 6.39 |
|  |  | 2008 | 763,439 | 44 | 5.76 |
|  |  | 2009 | 791,027 | 72 | 9.1 |
|  |  | 2010 | 818,695 | 28 | 3.42 |
|  |  | 2011 | 846,609 | 27 | 3.19 |
|  |  | 2012 | 874,520 | 24 | 2.74 |
|  |  | 2013 | 902,386 | 27 | 2.99 |
|  |  | 2014 | 930,165 | 36 | 3.87 |
|  |  | 2015 | 957,814 | 10 | 1.04 |
|  |  | 2016 | 985,498 | 23 | 2.33 |
| Magdalena | CRB | 2007 | 1,169,770 | 37 | 3.16 |
|  |  | 2008 | 1,180,051 | 32 | 2.71 |
|  |  | 2009 | 1,190,585 | 20 | 1.68 |
|  |  | 2010 | 1,201,386 | 32 | 2.66 |
|  |  | 2011 | 1,212,439 | 28 | 2.31 |
|  |  | 2012 | 1,223,781 | 27 | 2.21 |
|  |  | 2013 | 1,235,425 | 18 | 1.46 |
|  |  | 2014 | 1,247,383 | 24 | 1.92 |
|  |  | 2015 | 1,259,667 | 12 | 0.95 |
|  |  | 2016 | 1,272,278 | 11 | 0.86 |
| Meta | ORN | 2007 | 817,917 | 155 | 18.95 |
|  |  | 2008 | 835,461 | 795 | 95.16 |
|  |  | 2009 | 853,115 | 4019 | 471.1 |
|  |  | 2010 | 870,876 | 1591 | 182.69 |
|  |  | 2011 | 888,765 | 840 | 94.51 |
|  |  | 2012 | 906,755 | 1339 | 147.67 |
|  |  | 2013 | 924,843 | 1019 | 110.18 |
|  |  | 2014 | 943,024 | 1316 | 139.55 |
|  |  | 2015 | 961,292 | 1115 | 115.99 |
|  |  | 2016 | 979,683 | 636 | 64.92 |
| Nariño | PCF | 2007 | 1,580,123 | 347 | 21.96 |
|  |  | 2008 | 1,599,646 | 370 | 23.13 |
|  |  | 2009 | 1,619,464 | 989 | 61.07 |
|  |  | 2010 | 1,639,569 | 815 | 49.71 |
|  |  | 2011 | 1,660,087 | 598 | 36.02 |
|  |  | 2012 | 1,680,855 | 594 | 35.34 |
|  |  | 2013 | 1,701,840 | 704 | 41.37 |
|  |  | 2014 | 1,722,999 | 576 | 33.43 |
|  |  | 2015 | 1,744,275 | 442 | 25.34 |
|  |  | 2016 | 1,766,008 | 297 | 16.82 |
| Norte Santander | AND | 2007 | 1,265,006 | 169 | 13.36 |
|  |  | 2008 | 1,275,781 | 328 | 25.71 |
|  |  | 2009 | 1,286,728 | 249 | 19.35 |
|  |  | 2010 | 1,297,842 | 289 | 22.27 |
|  |  | 2011 | 1,309,217 | 132 | 10.08 |
|  |  | 2012 | 1,320,724 | 135 | 10.22 |
|  |  | 2013 | 1,332,335 | 164 | 12.31 |
|  |  | 2014 | 1,344,015 | 345 | 25.67 |
|  |  | 2015 | 1,355,723 | 466 | 34.37 |
|  |  | 2016 | 1,367,716 | 941 | 68.8 |
| Putumayo | AMZ | 2007 | 316,209 | 218 | 68.94 |
|  |  | 2008 | 319,390 | 315 | 98.63 |
|  |  | 2009 | 322,681 | 535 | 165.8 |
|  |  | 2010 | 326,093 | 402 | 123.28 |
|  |  | 2011 | 329,598 | 401 | 121.66 |
|  |  | 2012 | 333,247 | 421 | 126.33 |
|  |  | 2013 | 337,054 | 420 | 124.61 |
|  |  | 2014 | 341,034 | 353 | 103.51 |
|  |  | 2015 | 345,204 | 219 | 63.44 |
|  |  | 2016 | 349,537 | 118 | 33.76 |
| Quindío | AND | 2007 | 540,519 | 7 | 1.3 |
|  |  | 2008 | 543,532 | 12 | 2.21 |
|  |  | 2009 | 546,566 | 23 | 4.21 |
|  |  | 2010 | 549,624 | 24 | 4.37 |
|  |  | 2011 | 552,703 | 22 | 3.98 |
|  |  | 2012 | 555,806 | 9 | 1.62 |
|  |  | 2013 | 558,934 | 5 | 0.89 |
|  |  | 2014 | 562,087 | 6 | 1.07 |
|  |  | 2015 | 565,266 | 0 | 0 |
|  |  | 2016 | 568,473 | 2 | 0.35 |
| Risaralda | AND | 2007 | 908,654 | 183 | 20.14 |
|  |  | 2008 | 914,170 | 80 | 8.75 |
|  |  | 2009 | 919,653 | 133 | 14.46 |
|  |  | 2010 | 925,105 | 258 | 27.89 |
|  |  | 2011 | 930,523 | 110 | 11.82 |
|  |  | 2012 | 935,915 | 90 | 9.62 |
|  |  | 2013 | 941,283 | 290 | 30.81 |
|  |  | 2014 | 946,626 | 295 | 31.16 |
|  |  | 2015 | 951,945 | 295 | 30.99 |
|  |  | 2016 | 957,250 | 430 | 44.92 |
| San Andres | ISL | 2007 | 71,613 | 1 | 1.4 |
|  |  | 2008 | 72,167 | 0 | 0 |
|  |  | 2009 | 72,735 | 0 | 0 |
|  |  | 2010 | 73,320 | 0 | 0 |
|  |  | 2011 | 73,925 | 0 | 0 |
|  |  | 2012 | 74,541 | 0 | 0 |
|  |  | 2013 | 75,167 | 0 | 0 |
|  |  | 2014 | 75,801 | 0 | 0 |
|  |  | 2015 | 76,442 | 0 | 0 |
|  |  | 2016 | 77,101 | 0 | 0 |
| Santander | AND | 2007 | 1,979,090 | 610 | 30.82 |
|  |  | 2008 | 1,989,609 | 1145 | 57.55 |
|  |  | 2009 | 2,000,045 | 1252 | 62.6 |
|  |  | 2010 | 2,010,404 | 1082 | 53.82 |
|  |  | 2011 | 2,020,664 | 467 | 23.11 |
|  |  | 2012 | 2,030,857 | 524 | 25.8 |
|  |  | 2013 | 2,040,988 | 625 | 30.62 |
|  |  | 2014 | 2,051,065 | 1034 | 50.41 |
|  |  | 2015 | 2,061,095 | 553 | 26.83 |
|  |  | 2016 | 2,071,044 | 711 | 34.33 |
| Sucre | CRB | 2007 | 787,167 | 126 | 16.01 |
|  |  | 2008 | 794,904 | 199 | 25.03 |
|  |  | 2009 | 802,733 | 174 | 21.68 |
|  |  | 2010 | 810,650 | 80 | 9.87 |
|  |  | 2011 | 818,663 | 76 | 9.28 |
|  |  | 2012 | 826,755 | 116 | 14.03 |
|  |  | 2013 | 834,927 | 89 | 10.66 |
|  |  | 2014 | 843,182 | 59 | 7 |
|  |  | 2015 | 851,526 | 46 | 5.4 |
|  |  | 2016 | 859,909 | 24 | 2.79 |
| Tolima | AND | 2007 | 1,374,481 | 913 | 66.43 |
|  |  | 2008 | 1,378,937 | 297 | 21.54 |
|  |  | 2009 | 1,383,323 | 201 | 14.53 |
|  |  | 2010 | 1,387,641 | 1020 | 73.51 |
|  |  | 2011 | 1,391,890 | 438 | 31.47 |
|  |  | 2012 | 1,396,077 | 296 | 21.2 |
|  |  | 2013 | 1,400,203 | 676 | 48.28 |
|  |  | 2014 | 1,404,268 | 1176 | 83.74 |
|  |  | 2015 | 1,408,274 | 1082 | 76.83 |
|  |  | 2016 | 1,412,230 | 3224 | 228.29 |
| Valle | PCF | 2007 | 4,248,913 | 52 | 1.22 |
|  |  | 2008 | 4,293,230 | 304 | 7.08 |
|  |  | 2009 | 4,337,909 | 426 | 9.82 |
|  |  | 2010 | 4,382,939 | 495 | 11.29 |
|  |  | 2011 | 4,428,342 | 235 | 5.31 |
|  |  | 2012 | 4,474,084 | 241 | 5.39 |
|  |  | 2013 | 4,520,166 | 155 | 3.43 |
|  |  | 2014 | 4,566,593 | 148 | 3.24 |
|  |  | 2015 | 4,613,377 | 145 | 3.14 |
|  |  | 2016 | 4,660,438 | 137 | 2.94 |
| Vaupés | AMZ | 2007 | 40,198 | 7 | 17.41 |
|  |  | 2008 | 40,649 | 17 | 41.82 |
|  |  | 2009 | 41,094 | 42 | 102.2 |
|  |  | 2010 | 41,534 | 113 | 272.07 |
|  |  | 2011 | 41,965 | 429 | 1022.28 |
|  |  | 2012 | 42,392 | 445 | 1049.73 |
|  |  | 2013 | 42,817 | 53 | 123.78 |
|  |  | 2014 | 43,240 | 115 | 265.96 |
|  |  | 2015 | 43,665 | 38 | 87.03 |
|  |  | 2016 | 44,079 | 21 | 47.64 |
| Vichada | ORN | 2007 | 58,885 | 20 | 33.96 |
|  |  | 2008 | 60,494 | 29 | 47.94 |
|  |  | 2009 | 62,071 | 37 | 59.61 |
|  |  | 2010 | 63,670 | 91 | 142.92 |
|  |  | 2011 | 65,282 | 120 | 183.82 |
|  |  | 2012 | 66,917 | 154 | 230.14 |
|  |  | 2013 | 68,575 | 70 | 102.08 |
|  |  | 2014 | 70,260 | 106 | 150.87 |
|  |  | 2015 | 71,974 | 119 | 165.34 |
|  |  | 2016 | 73,702 | 16 | 21.71 |
